# Evaluation of Cytokines as Robust Diagnostic Biomarkers for COVID-19 Detection

**DOI:** 10.3390/jpm11070681

**Published:** 2021-07-20

**Authors:** Álvaro Tamayo-Velasco, María Jesús Peñarrubia-Ponce, Francisco Javier Álvarez, Hugo Gonzalo-Benito, Ignacio de la Fuente, Marta Martín-Fernández, José María Eiros, Pedro Martínez-Paz, José Pablo Miramontes-González, Aida Fiz-López, Elisa Arribas-Rodríguez, Paloma Cal-Sabater, Rocío Aller, Carlos Dueñas, María Heredia-Rodríguez, Eduardo Tamayo, David Bernardo, Esther Gómez-Sánchez

**Affiliations:** 1 Haematology and Hemotherapy Department, Hospital Clínico Universitario de Valladolid, 47003 Valladolid, Spain; alvarotv1993@gmail.com (Á.T.-V.); mpenarrubia@saludcastillayleon.es (M.J.P.-P.); ifuentegr@saludcastillayleon.es (I.d.l.F.); 2 Pharmacological Big Data Laboratory, Pharmacology Department, Faculty of Medicine, Universidad de Valladolid, 47005 Valladolid, Spain; alvarez@med.uva.es; 3 BioCritic, Group for Biomedical Research in Critical Care Medicine, 47005 Valladolid, Spain; hgonzalob@saludcastillayleon.es (H.G.-B.); pedrojose.martinez@uva.es (P.M.-P.); rallerf@saludcastillayleon.es (R.A.); maria_her_05@hotmail.com (M.H.-R.); eduardo.tamayo@uva.es (E.T.); esthergzam@hotmail.com (E.G.-S.); 4 Research Unit, Hospital Clínico Universitario de Valladolid, 47003 Valladolid, Spain; 5 Institute of Health Sciences of Castile and Leon (IECSCYL), 47002 Soria, Spain; 6 Department of Medicine, Dermatology and Toxicology, Faculty of Medicine, Universidad de Valladolid, 47005 Valladolid, Spain; 7 Microbiology Department, Hospital Universitario Rio Hortega, 47012 Valladolid, Spain; jmeirosbouza@gmail.com; 8 Department of Surgery, Faculty of Medicine, Universidad de Valladolid, 47005 Valladolid, Spain; 9 Instituto de Investigación Biomédica de Salamanca (IBSAL), Universidad Pontificia de Salamanca, 37002 Salamanca, Spain; jpmiramontes@hotmail.com; 10 Internal Medicine Department, Hospital Universitario Rio Hortega, 47012 Valladolid, Spain; 11 Mucosal Immunology Laboratory, Instituto de Biomedicina y Genética Molecular (IBGM), Universidad de Valladolid-CSIC, 47005 Valladolid, Spain; aidafizlopez@gmail.com (A.F.-L.); elisarribas@usal.es (E.A.-R.); calsabater89@gmail.com (P.C.-S.); d.bernardo.ordiz@gmail.com (D.B.); 12 Gastroenterology Department, Hospital Clínico Universitario de Valladolid, 47003 Valladolid, Spain; 13 Internal Medicine Department, Hospital Clínico Universitario de Valladolid, 47003 Valladolid, Spain; carlos.duenas@hotmail.com; 14 Anesthesiology and Critical Care Department, Hospital Clínico Universitario de Salamanca, 37007 Salamanca, Spain; 15 Anesthesiology and Critical Care Department, Hospital Clínico Universitario de Valladolid, 47003 Valladolid, Spain; 16 Centro de Investigación Biomédica en Red de Enfermedades Hepáticas y Digestivas (CIBERehd), 28029 Madrid, Spain

**Keywords:** COVID-19, IP-10, diagnosis, biomarker, validation

## Abstract

Antigen tests or polymerase chain reaction (PCR) amplification are currently COVID-19 diagnostic tools. However, developing complementary diagnosis tools is mandatory. Thus, we performed a plasma cytokine array in COVID-19 patients to identify novel diagnostic biomarkers. A discovery–validation study in two independent prospective cohorts was performed. The discovery cohort included 136 COVID-19 and non-COVID-19 patients recruited consecutively from 24 March to 11 April 2020. Forty-five cytokines’ quantification by the MAGPIX system (Luminex Corp., Austin, TX, USA) was performed in plasma samples. The validation cohort included 117 patients recruited consecutively from 15 to 25 April 2020 for validating results by ELISA. COVID-19 patients showed different levels of multiple cytokines compared to non-COVID-19 patients. A single chemokine, IP-10, accurately identified COVID-19 patients who required hospital admission (AUC: 0.962; 95%CI (0.933–0.992); *p* < 0.001)). The results were validated in an independent cohort by multivariable analysis (OR: 25.573; 95%CI (8.127–80.469); *p* < 0.001) and AUROC (AUC: 0.900; 95%CI (0.846–0.954); *p* < 0.001). Moreover, showing IP-10 plasma levels over 173.35 pg/mL identified COVID-19 with higher sensitivity (86.20%) than the first SARS-CoV-2 PCR. Our discover–validation study identified IP-10 as a robust biomarker in clinical practice for COVID-19 diagnosis at hospital. Therefore, IP-10 could be used as a complementary tool in clinical practice, especially in emergency departments.

## 1. Introduction

A new strain of coronavirus, severe acute respiratory syndrome coronavirus 2 (SARS-CoV-2), was recognized in December 2019 in China. Since then, more than 140 million cases have been reported, causing more than 3,000,000 deaths [1]. The incubation time is approximately 5 days [2]. Clinical presentation is broad. Most common symptoms include fever, cough and fatigue. However, it can also be presented from a totally asymptomatic infection to an acute respiratory distress syndrome with multiorgan dysfunction [1,3,4].

Hospitalized patients usually present moderate or severe respiratory disease with high fever and pneumonia [5]. Certain biomarkers [6,7] were related to this situation, as well as radiological alterations [8,9]. Nevertheless, clinical, radiological and laboratory features cannot be easily distinguished from pneumonia induced by other viral or bacterial infections [10].

Precise and early diagnosis is the cornerstone for both optimal prevention and treatment, especially in hospitals. Polymerase chain reaction (PCR) is the main diagnostic test for COVID-19, but its sensitivity is variable [11], reaching a 71% sensitivity in some reports [12]. This can be due to an inappropriate sample taking [13], hence demanding new PCR tests performance if a negative result occurred and a high probability of infection existed [12]. This translates into a delay in diagnosis and treatment options, essential not just for patient survival but also in preventive quarantine and hospital organization [14].

Although several logistic prediction models were recently developed, none of them were entirely effective for their implementation into clinical practice [15,16,17,18,19]. Although many models showed optimal area under the curve (AUC), they are complex to use in clinical routine. Considering that cytokine profile could play an important role in the pathogenesis of COVID-19 hospitalized patients, various studies suggested the association of certain cytokines with an unfavorable prognosis in this disease [20,21,22]. Hence, it would be useful to identify a simple, accurate and specific biomarker to detect COVID-19 patients in order to make prompt decisions until a final confirmation is made or even avoiding the necessity of PCR tests performance. In this regard, a similar approach with the use of troponin T in acute myocardial infarction or procalcitonin (PCT) in sepsis was developed.

In this work, we aid in identifying a sensible and specific diagnostic biomarker of COVID-19 disease by prospectively assessing plasma levels of 45 cytokines and growth factors aiming to allow early detection of COVID-19 patients, especially in emergency rooms.

## 2. Materials and Methods

### 2.1. Study Design and Patient Selection

A prospective study was designed with two different cohorts: (i) A discovery cohort to identify biomarkers that were able to classify patients in COVID-19 and non-COVID-19; (ii) A validation cohort that included different COVID-19 and non-COVID-19 patients to confirm the results. 

The discovery cohort was prospective and included 136 consecutive patients older than 18 years admitted at the Hospital Clínico Universitario de Valladolid (Valladolid, Spain) between 24 March and 11 April 2020. A total of 108 patients were positive for COVID-19 as confirmed by polymerase chain reaction (PCR) of a nasopharyngeal sample immediately after hospital admission. Patients with concomitant infections and/or any chronic terminal illnesses were excluded from the study. The remaining 28 patients were admitted to the hospital in the same time period for elective major surgery with a negative PCR result for SARS-CoV-2 infection. The validation cohort included 117 consecutive patients older than 18 years, recruited between 15 and 25 April 2020 at the emergency room of the same hospital. Of them, 58 patients were PCR-diagnosed with COVID-19, while 59 were PCR negative for SARS-CoV-2 in at least two nasopharyngeal samples.

### 2.2. Biological Samples

Blood samples from the discovery cohort were collected in the first 24 h from hospital admission at 09:00 h using 3.2% sodium citrate tubes and centrifuging them at 2000× *g* for 20 min at room temperature, while blood samples from the validation cohort were collected when the patients attended the emergency admission room at the hospital. In both cases, plasma was obtained, aliquoted and cryopreserved at −80 °C until used. All the samples were relabeled with an internal laboratory number to guarantee blindness and randomly distributed to avoid biases in the experimental procedures.

### 2.3. Cytokines and Chemokines Analysis and Interpretation

Plasma samples from patients of the discovery cohort were measured by a duplicate for each patient using a MAGPIX system (Luminex). Forty-five protein targets were analyzed with the Cytokine/Chemokine/Growth Factor 45-Plex Human ProcartaPlex™ Panel 1 (Invitrogen, Waltham, MA, USA) following the manufacturer’s guidelines and recommendations. Cytokines, chemokines and growth factors included in the panel were BDNF, EGF, Eotaxin (also known as CCL11), FGF-2, GM-CSF, GRO-α (CXCL1), HGF, IFN-α, IFN-γ, IL-1α, IL-1β, IL-10, IL-12p70, IL-13, IL-15, IL-17A (CTLA-18), IL-18, IL-1RA, IL-2, IL-21, IL-22, IL-23, IL-27, IL-31, IL-4, IL-5, IL-6, IL-7, IL-8 (also known as CXCL8), IL-9, IP-1 beta (CCL4), IP-10 (CXCL10), LIF, MCP-1 (CCL2), MIP-1α (CCL3), NGF-β, PDGF-BB, PIGF-1, RANTES (CCL5), SCF, SDF-1α, TNFα, TNFβ, VEGF-A and VEGF-D.

For cytokine interpretation, cytokine values below the detection limit were imputed by using robust regression on order statistics. This method performs a regression to impute low values assuming log-normal quantiles for samples with a detection rate of at least 30% after checking that the data follow a log-normal distribution. To accomplish this, the non-detects and data analysis (NADA) R package was used. Molecules detected in less than 30% of the samples (FGF-2, IL-12, IL-21, IL-23, IL-31, IL-9, NGFβ and TNFβ) were not statistically analyzed any further. Cytokine expression data were transformed using the logarithmic base 2 scale. 

Plasma samples from the validation cohort were analyzed with the IP-10 (CXCL10) Human ELISA Kit (Invitrogen, Waltham, MA, USA) following the manufacturer’s instructions.

### 2.4. Variables

Demographic (age and gender), clinical (use of tobacco, use of alcohol, hypertension, cardiac disease, diabetes, neurological disease, liver disease, obesity, lung disease and kidney disease) and analytical data (glycemia, creatinine, total bilirubin, leukocytes, lymphocytes, neutrophils, platelets, C-reactive protein, procalcitonin, ferritin and D dimer) of each patient were recorded to describe the clinical phenotype. 

### 2.5. Ethics Approval

The study was approved by the hospital’s Clinical Ethics Committee (cod: PI 20-1716 and PI 20-1717). All patients provided informed consent to be included in the study. It followed the code of ethics of the World Medical Association (Declaration of Helsinki). 

### 2.6. Statistical Analysis

Demographic and clinical characteristics were evaluated by using the Chi-square test for categorical variables and the Mann–Whitney U test for continuous variables when appropriate. The median values and interquartile range (IR) were used to describe quantitative variables. The percentage and total number were used for categorical ones.

We estimated the diagnostic accuracy of cytokines and other biomarkers to identify COVID-19 patients by using the area under the receiver operator characteristic curve (AUROC). The optimal operating point (OOP) of IP-10 was calculated, being the value for which the point on the curve had the minimum distance to the upper left corner (where sensitivity = 1 and specificity = 1). By Pythagoras’ theorem, this distance is Optimal Operating Point (OOP) = √(1−sensitivity)2 + (1−specificity)2. 

Individual logistic regression models, in which the exponential of the coefficients can be directly interpreted as odds ratio, were applied. A backward stepwise multivariate logistic regression analysis was performed to assess independent associations with the diagnosis of the COVID-19 disease. The multivariate model used all those markers that showed a certain degree of association with the disease. A cut-off point < 0.1 in the *p*-value was required in the individual model. Internal validation tried to determine the discrimination capacity of the model and its capacity to classify new individuals correctly. As a global measure, we use the AU-ROC, knowing that a model is a perfect classifier when the AUC is 1.

Statistical analysis was performed using the R statistical package version 3.1.1 (The R Foundation, Vienna, Austria) and also the statistical package SPSS statistics software (SPSS) version 25 (IBM, Armonk, NY, USA). The statistical significance was set at *p* ≤ 0.05.

## 3. Results

### 3.1. Demographics

A total of 253 patients were registered in the study and divided into two groups: discovery cohort (*n* = 136) and validation cohort (*n* = 117). The clinical characteristics of both cohorts are shown in Table 1. COVID-19 and non-COVID-19 were similar in terms of gender in both cohorts. In terms of age, there were no differences in discovery cohort, (69 (20) vs. 71 (37), *p* = 0.296) although COVID-19 patients were significantly older compared to non-COVID-19 controls in the validation one (77.5 (19) vs. 59 (36), *p* < 0.001).

Both cohorts did not show statistical differences in any comorbidities. COVID-19 and non-COVID-19 patients associated hypertension as principal comorbidity followed by the presence of diabetes, lung disease or coronary disease. Compared to laboratory assessments, COVID-19 patients in both cohorts had significantly lower lymphocyte count as well as higher C-reactive protein and D-dimer. Both lower platelet and leukocyte count, as well as higher neutrophils levels, were observed only in the discovery cohort. The length of hospital stay was similar in COVID-19 patients, showing a median of 12 days in the discovery cohort and a median of 11.5 days in the validation one. Non-COVID-19 patients associated significantly lower in-hospital stay. Finally, 18.5% and 14% percentage 28-day mortality were found in COVID-19 patients in the discovery and validation cohort, respectively, while no deaths were recorded across any cohort in non-COVID-19 patients.

### 3.2. Discovery Cohort

**Figure 1:**
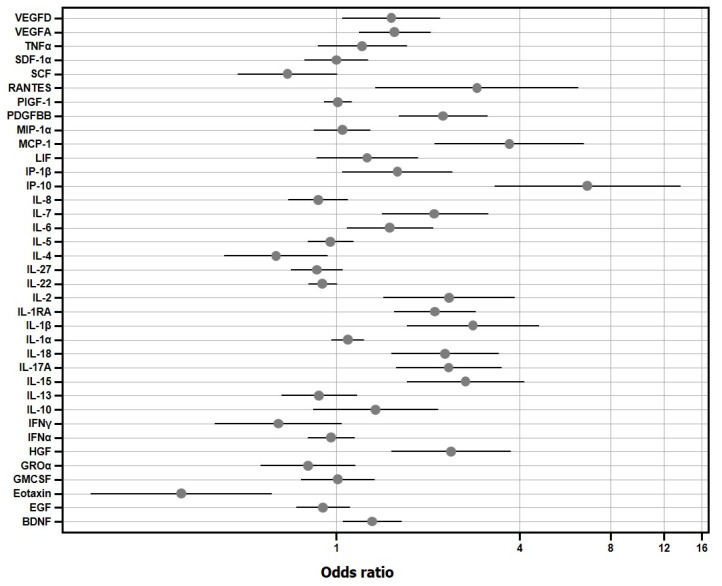
Individual logistic regression models showing odds ratio and 95% confidence interval for each cytokine. Note the logarithmic x-axis.

Plasma levels of 24 cytokines showed statistically significant differences between COVID-19 and non-COVID-19 patients (Table S1). The individual logistic regression models, adjusting by age and gender, showed statistically significant overexpression of 17 cytokines (BDNF, HGF, IL-1β, IL-15, IL-17A, IL-18, IL-1RA, IL-2, IL-6, IL-7, IP1b, IP-10, MCP1, PDGFBB, VEGFA, VEGFD, RANTES) in COVID-19 patients, while IFN γ, IL-22, IL-4 and SCF were under-expressed (Figure 1).

Based on these results, we performed the area under the ROC curve (AUC) analysis to determine the accuracy of each cytokine to classify COVID-19 patients properly. A total of -8 molecules showed a significant result in AUC analysis (Table 2). However, only IL-17a, MCP1, PDGFBB, IL1RA and IP-10 showed an AUC over 0.80. Indeed, IP-10 was the best biomarker for early COVID-19 diagnosis, reaching an AUC of 0.95.

### 3.3. Validation Cohort

**Figure 2:**
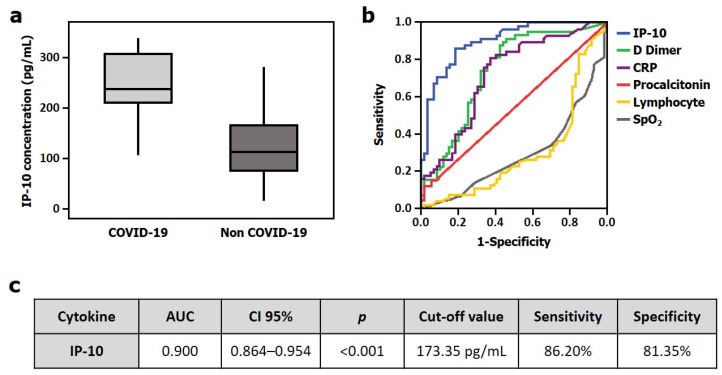
IP-10 in the validation cohort. (**a**) IP-10 plasma levels between COVID-19 and non-COVID-19 patients (Mann–Whitney U test). (**b**) Area under the ROC curve (AUC) analysis for evaluating the accuracy of different parameters for detecting COVID-19 infection. (**c**) Sensitivity and specificity calculation for IP-10 cut-off point.

Taking into account the results elucidating the potential role of IP-10 as a diagnostic biomarker in the COVID-19 disease, we next aimed to validate these findings in a second independent cohort. Hence, the IP-10 median plasma levels in this second cohort were higher in COVID-19 patients (238.67 (100)) compared to non-COVID-19 (112.18 (93)) controls (Figure 2a). Next, we compared IP-10 accuracy for COVID-19 detection with other biomarkers widely recognized for identifying infection such as C reactive protein or procalcitonin as well as other parameters related to the COVID-19 disease such as D dimer, SpO*_2_* and lymphocyte count. All parameters, except procalcitonin, displayed significant utility for COVID-19 diagnosis (Table S2). However, IP-10 was the only one presenting an AUC over 0.70, revealing it as the best biomarker to detect COVID-19 (AUC = 0.900 (95% CI [0.846–0.954], *p* < 0.001); Figure 2b). Moreover, the resulting cut-off value of IP-10 was 173.35 pg/mL, which showed a sensitivity of 86.20% and specificity of 81.35% for identifying COVID-19 infection (Figure 2c).

A multivariate model was performed with an automatic logistic regression by back steps, including IP-10 > 173.35 pg/mL together with age, hypertension, creatinine, lymphocyte counts, D Dimer and CRP (Table 3) as adjusting variables. The IP-10 levels above its cut-off value were independently associated with COVID-19 disease diagnosis. In fact, patients showing IP-10 levels over 173.35 pg/mL presented a 25-fold risk of COVID-19 infection (OR: 25.573; 95% CI (8.127–80.469); *p* < 0.001). The internal validation of this multivariate model by the LOOCV procedure was performed using the R statistical package version 3.1.1 (The R Foundation, Vienna, Austria) and confirmed the previous results (AUC = 0.902, CI 95% (0.846–0.958), *p* < 0.001).

**Figure 3:**
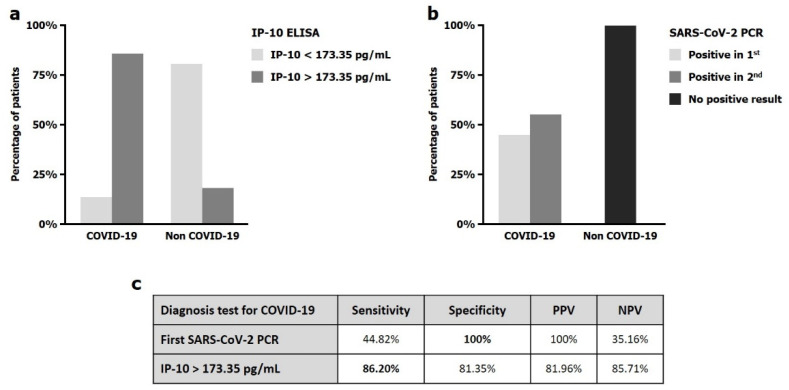
Histograms and table showing sensitivity and specificity diagnosis of IP-10 cut-off point (**a**) and first SARS-CoV-2 PCR (**b**). Sensitivity, specificity, positive predictive value (PPV) and negative predictive value (NPV) for first SARS-CoV-2 PCR and IP-10 ELISA (**c**).

The IP-10 cut-off value showed a great balance between sensitivity (86.20%) and specificity (81.35%) for COVID-19 diagnosis (Figure 3). Thus, we compared it with the diagnostic ability of the first PCR for SARS-CoV-2 detection (Figure 3). Our results elucidated that the sensitivity of the first SARS-CoV-2 PCR in the emergency room is limited (44.82%), indicating that more than 50% of COVID-19 patients had a negative result in the first PCR, while IP-10 screening and early diagnosis capacity reached almost 90% (Table S3).

## 4. Discussion

A simple and precise biomarker in the clinical practice for early COVID-19 disease in the emergency room before hospital admission would have a direct and immediate impact. In this regard, we characterized the plasma cytokine profile of COVID-19 patients aiming to identify and validate novel biomarkers complementary to PCR or antigen tests. Hence, we hereby have shown how IP-10 is statistically increased in COVID-19 patients at hospital admission compared to non-COVID-19 patients. Indeed, IP-10 identified COVID-19 patients who require hospital admission with great accuracy (AUC: 0.962; *p* < 0.001; 95% CI (0.933–0.992)), improving the diagnostic capacity of classical biomarkers. Last but not least, IP-10 plasma levels over 173.35 pg/mL were more sensitive (86.20%) than the first SARS-CoV-2 PCR (44.82%) to identify COVID-19 patients susceptible to be admitted to the hospital.

An early and appropriate identification and isolation of potential COVID-19 patients is essential to enable timely treatment, conserve resources, protect patients and healthcare personnel and prevent the spread of the infection in healthcare facilities [1,4]. Until now, nasopharyngeal sampling, either for PCR or antigen tests, was widely used for this task. Nevertheless, the relative unreliability [11] in detecting upper respiratory viruses (30% negative results) [12] due to immature development of nucleic acid detection technology, variation in the detection rates of different manufacturers, prolonged nucleic acid conversion [23], inadequate clinical sampling or presence of low viral load in samples [13], as well as the delays [24] in obtaining results bring to light the necessity of developing of alternative solutions. In this regard, we demonstrated the same difficulties in addition to low sensitivity (44.82% of first SARS-CoV-2 PCR).

Based on radiological manifestations, the use of chest Computed Tomography (CT) for a reliable and early diagnosis was studied [25], displaying a shorter delay in obtaining results in comparison to PCR [24]. However, the sensitivity and accuracy of CT are variable [24,25,26], and also the diagnosis in patients showing minimal symptoms is limited and requires the transfer inside the hospital, associating risk of infection spread. In the same way, trying to improve the diagnosis of COVID-19, several logistic prediction models were developed during the last months [16,17,18,27]. However, these models are not validated, and their diagnostic accuracy is limited, including subjective variables making its implementation in clinical routine difficult [19]. Furthermore, the quick advance and evolution of the pandemic around the world make them outdated. In order to overcome these limitations, we hereby focused on the study of several plasma immune mediators, which can be accurately determined in an automatized manner, keeping in mind that COVID-19 severity was linked to abnormal cytokine and chemokines plasma levels [28,29,30]. We not only confirmed the overexpression and underexpression of multiple cytokines in COVID-19 patients, but we also found IP-10 as an early robust and validated diagnosis biomarker in the COVID-19 disease.

IP-10 (CXCL10) is a small 10.8kD protein secreted by many cell types in response to interferon-gamma (IFNγ) [31]. Hence, it may be a potential marker for lung diseases due to its known role as an important mediator for recruiting activated lymphocytes into the lungs [32]. In fact, IP-10 has been related to M. tuberculosis [33], H5N1, H1N1 [34], SARS-CoV, MERS-CoV or T-cell alveolitis [31,35,36]. Regarding the potential implication of IP-10 in the pathogenesis of SARS-CoV-2, Yu Chen et al. reported the association between IP-10 and MCP-1 levels and disease severity of COVID-19 [31]. Yang et al. also related IP-10 to disease severity and progression [35]. In this sense, taking into account the potential role of IP-10 in ARDS pathology [37], an anti-IP-10 antibody was proposed as a possible treatment in COVID-19 patients [31]. It seems to be logical to associate high levels of IP-10 only in COVID-19 patients with respiratory symptoms and normal IP-10 levels in asymptomatic ones, as our study confirmed.

Our study also associated unicentric recruitment of both cohorts as a limitation. However, we performed a discovery–validation study in two independent prospective cohorts that confirmed IP-10 levels measured by ELISA as a robust and novel biomarker in clinical practice. Therefore, the clinical use of IP-10 could be similar to that of troponin T for acute myocardial infarction or PCT for sepsis. Quick and easy display and interpretation make IP-10 feasible for clinical implementation as a COVID-19 diagnostic tool, especially in emergency rooms.

## 5. Conclusions

A discovery–validation study in two independent prospective cohorts to describe the plasma cytokine profile of COVID-19 patients confirmed that the determination of IP-10 plasma levels by ELISA could be a novel and robust biomarker of COVID-19 disease in the clinical practice. Indeed, IP-10 plasma levels above 173.35 pg/mL were significantly more sensitive than the first SARS-CoV-2 PCR to identify COVID-19 patients suitable for hospital admission. Hence, IP-10 shows a great balance between sensitivity and specificity to identify COVID-19. In summary, IP-10 determination would be useful to identify in a simple, accurate and specific manner COVID-19 patients in order to make prompt decisions until a final PCR confirmation is made or even avoiding the necessity of PCR tests performance, making it a valuable candidate for its implementation in clinical routine, especially in the emergency department.

## 6. Patents

This approach has already been registered as a European patent: “Method for the diagnosis of a coronavirus infection”, Number: EP21382219.0, March 2021.

## Figures and Tables

**Table 1:** Clinical characteristics of COVID-19 and non-COVID-19 patients in both the discovery cohort and validation cohort. Continuous variables are represented as median, (interquartile range, IQR); categorical variables are represented as %, (*n*). CRP, C-reactive protein. The bold is to indicate statistical significance.

	Discovery Cohort			Validation Cohort		
	COVID-19 (*n* = 108)	NON-COVID-19 (*n* = 28)	*p*	COVID-19 (*n* = 58)	NON-COVID-19 (*n* = 59)	*p*
Age (years; median (IQR))	69 (20)	71 (37)	0.296	77.5 (19)	59 (36)	**<0.001**
Male (%(*n*))	55.3 (73)	57.1 (16)	0.722	44.8 (26)	44.1 (26)	0.934
**-Comorbidities (%(*n*))**						
Use of tobacco	8.3 (9)	10.7 (3)	0.692	1.7 (1)	10.2 (6)	0.154
Use of alcohol	2.8 (3)	7.1 (2)	0.274	1.7 (1)	5.1 (3)	0.317
Hypertension	46.3 (50)	50 (14)	0.492	50 (29)	28.8 (17)	**0.019**
Cardicac disease	13. 0 (14)	21.4 (6)	0.815	13.8 (8)	8.5 (5)	0.360
Diabetes	17.6 (19)	14.3 (4)	0.677	5.2 (3)	11.9 (7)	0.195
Neurological disease	2.8 (3)	3.6 (1)	0.974	6.9 (4)	6.8 (4)	0.980
Liver disease	1.9 (2)	3.6 (1)	0.581	0 (0)	3.4 (2)	0.157
Obesity	9.3 (10)	10.7 (3)	0.770	10.3 (6)	5.1 (3)	0.286
Lung disease	16.8 (18)	14.3 (4)	0.944	6.9 (4)	10.2 (6)	0.527
Kidney disease	2.8 (3)	3.6 (1)	0.825	0 (0)	0 (0)	-
**-Laboratory (median (IQR))**						
Glycaemia (mg/dL)	123 (82)	94.6 (12)	0.051	109 (28)	109 (24)	0.391
Creatinine (mg/dL)	0.84 (0.36)	0.8 (0.35)	0.495	0.87 (0.63)	0.78 (0.35)	**0.030**
Total bilirubin (mg/dL)	0.5 (0.34)	0.3 (0.4)	0.06	0.27 (0.2)	0.27 (0.21)	0.696
Leukocytes (×10^9^/L)	6.68 (3.13)	6.87 (2.15)	**<0.001**	8.11 (6.80)	7.76 (4.35)	0.482
Lymphocytes (×10^9^/L)	1.09 (0.90)	2.25 (0.94)	**<0.001**	1.14 (0.67)	1.6 (0.81)	**<0.001**
Neutrophil (×10^9^/L)	4.46 (3.22)	3.76 (1.34)	**<0.001**	5.18 (5.13)	4.69 (3.59)	0.821
Platelet (×10^9^/L)	218 (108)	250 (580)	**0.005**	203 (67)	203 (64)	0.839
D-dimer (ng/mL)	742 (1267)	255 (106)	**<0.001**	258 (976)	992 (1860)	**<0.001**
CRP (mg/L)	80 (120)	10 (3)	**<0.001**	32.9 (99.75)	3.3 (50.8)	**<0.001**
Procalcitonin (ng/mL)	0.13 (0.26)	0.11 (0.1)	0.323	0.01 (0)	0.01 (0)	0.185
**-Hospital meters (median (IQR))**						
Length of hospital stay	12 (13)	4.50 (3)	**<0.001**	11.5 (15.5)	7 (1)	0.320
**-Mortality (%(*n*))**						
28-day mortality	18.5 (20)	0 (0)	**<0.001**	24.1 (14)	0 (0)	**<0.001**

**Table 2:** Area under the receiver operating characteristic curve (AUROC) analysis of the 18 significant cytokines. The bold is to indicate statistical significance.

	CYTOKINE	AUC	CI 95%	*p*
1	IP-10	**0.962**	**0.933–0.992**	**<0.001**
2	IL1RA	0.866	0.795–0.936	<0.001
3	PDGFBB	0.866	0.800–0.931	<0.001
4	MCP1	0.839	0.767–0.911	<0.001
5	IL17a	0.800	0.718–0.882	<0.001
6	IL15	0.792	0.703–0.880	<0.001
7	IL1b	0.790	0.710–0.869	<0.001
8	HGF	0.787	0.700–0.874	<0.001
9	IL18	0.787	0.701–0.873	<0.001
10	IL7	0.758	0.681–0.836	<0.001
11	IL2	0.723	0.639–0.807	<0.001
12	VEGFA	0.705	0.591–0.818	0.001
13	RANTES	0.704	0.603–0.806	0.001
14	VEGFD	0.671	0.538–0.804	0.005
15	IP1b	0.666	0.548–0.748	0.060
16	IL6	0.659	0.572–0.746	0.045
17	INF	0.329	0.234–0.423	0.048
18	IL4	0.321	0.223–0.420	0.050

**Table 3:** Multivariate model showing the association between high IP-10 levels and risk of COVID-19 infection. CI, confidence interval; OR, odds ratio; CRP, C reactive protein. The bold is to indicate statistical significance.

		OR	CI 95%	*p*
COVID-19 disease	IP-10 > 173.35 pg/mL	**25.573**	**8.127–80.469**	**<0.001**
	Age	1.011	0.977–1.047	0.528
	Hypertension	0.806	0.211–3.081	0.753
	Creatinine	1.507	0.608–3.739	0.376
	Lymphocytes	0.999	0.999–1.000	0.235
	D Dimer	1.000	1.000–1.000	0.191
	CRP	1.001	0.993–1.008	0.897

## Data Availability

The datasets during and/or analyzed during the current study are available from the corresponding author on reasonable request.

## Supplementary Materials

The following are available online at https://www.mdpi.com/article/10.3390/jpm11070681/s1, Table S1: Level of cytokine comparing cases and control. They are represented as median (interquartile range, IQR) in Table S2: Area under the receiver operating characteristic curve (AUROC) analysis of IP-10 compared to other biomarkers, Table S3: Sensitivity and specificity diagnosis of first PCR SARS-CoV-2 (a) and IP-10 cut-off point (b).
