# Supplementary material for: Evaluation of Cytokines as Robust Diagnostic Biomarkers for COVID-19 Detection
Source: J Pers Med. 2021 Jul 20;11(7):681. doi: 10.3390/jpm11070681 (PMC8303564; doi:10.3390/jpm11070681)
Supplement: Supplementary file 1 [file jpm-11-00681-s001.zip › jpm-1283626-supplementary.pdf]

Cytokine	Control N = 28		Cases N = 108		*p*
	Median	IQR	Median	IQR	
BDNF	39.01	115.95	58.67	132.52	**0.024**
EGF	3.78	1.48	2.15	7.4	0.171
Eotaxin	23.82	12.66	13.85	9.6	**<0.001**
GMCSF	15.07	6.19	11.38	25.39	0.733
GROα	4.26	1.83	2.99	4.17	0.190
HGF	71.45	53.6	163.75	251.66	**<0.001**
IFNα	0.74	0.37	0.48	1.63	0.499
IFNg	12.56	3.4	8.81	6.7	**0.005**
IL-1α	0.58	0.56	2.5	7.76	**0.012**
IL-1β	2.49	0.96	6.62	10.47	**<0.001**
IL-10	1.81	0.5	1.69	2.54	0.611
IL-13	3.17	1.5	2.06	2.86	0.093
IL-15	4.41	2.96	13.32	17.99	**<0.001**
IL-17A	2.03	0.61	7.03	14.73	**<0.001**
IL-18	14.53	18.43	47.2	51.74	**<0.001**
IL-1RA	72.51	38.73	604.75	1007.92	**<0.001**
IL-2	7.59	1.61	13.39	19.64	**<0.001**
IL-22	18.15	22.33	3.57	20.89	**0.015**
IL-27	38.87	18.2	18.06	34.08	**0.023**
IL-4	10.37	4.52	5.59	6.98	**0.04**
IL-5	9.42	4.88	5.12	17.34	0.394
IL-6	8.69	3.56	13.07	22.73	**0.01**
IL-7	0.72	0.31	1.67	2.88	**<0.001**
IL-8	3.25	1.69	2	3.81	0.079
IP1b	29.95	22.42	48.6	44.16	**0.007**
IP-10	5.56	4.2	45.67	40.46	**<0.001**
LIF	12.18	2.46	14.9	16.81	0.105

Cytokine	Control N = 28		Cases N = 108		*p*
	Median	IQR	Median	IQR	
MCP1	16.78	14.57	37.88	31.55	**<0.001**
MIP1a	2.91	1.12	3.39	11.38	0.607
PDGF-BB	31.8	39.64	303.42	656.93	**<0.001**
PIGF1	5.82	5.16	5.45	60.52	0.807
RANTES	17.62	8.36	22.77	18.9	**0.001**
SCF	9.52	12.13	6.51	7.49	0.199
SDF1a	951.75	412.62	669.5	607.62	**0.011**
TNFα	6.46	3.29	6.02	10.91	0.541
VEGFA	47.8	98.36	122.65	188.12	**0.001**
VEGFD	6.55	10.74	12.65	13.28	**0.005**

Table S1: Level of cytokine comparing cases and control. They are represented as [median. (interquartile range. IQR)].

Parameter	AUC	*p*	CI 95%
**IP-10**	0.900	<0.001	0.846 – 0.954
**D Dimer**	0.736	<0.001	0.643 – 0.828
**SpO2**	0.289	<0.001	0.195 – 0.382
**CRP**	0.717	<0.001	0.624 – 0.811
**Lymphocytes**	0.300	<0.001	0.202 – 0.398
**Procalcitonin**	0.537	0.485	0.433 – 0.642

Table S2: Area under the receiver operating characteristic curve (AUROC) analysis of IP-10 compared to other biomarkers.

a	COVID-19	Non COVID-19		
**First PCR SARS-CoV-2: Positive**	26 patients	0 patients	26 patients	**PPV 100%**
**First PCR SARS-CoV-2: Negative**	32 patients	59 patients	91 patients	**NPV 35.16%**
	58 patients	59 patients	117 patients	
	**Sensitivity 44.82%**	**Specificity 100%**		

b	COVID-19	Non COVID-19		
**IP-10 > 173.35 pg/mL**	50 patients	11 patients	61 patients	**PPV 81.96%**
**IP-10 < 173.35 pg/mL**	8 patients	48 patients	56 patients	**NPV 85.71%**
	58 patients	59 patients	117 patients	
	**Sensitivity 86.20%**	**Specificity 81.35%**		

Table S3: Sensitivity and specificity diagnosis of first PCR SARS-CoV-2 (a) and IP-10 cut-off point (b).
